# Breaking the Triple “Skeleton–Liquid Loading–Liquid Lock” Barrier: Research Progress and Integrated Prospects for Surfactants for Repairing Deep Natural Gas Reservoirs

**DOI:** 10.3390/molecules31213906

**Published:** 2026-10-29

**Authors:** Dongxiao Xu, Dingfan Xue, Jingjing Jiang, Jian Guo, Naipeng Zhao, Yubo Lian, Gang Chen

**Affiliations:** 1 Xi’an Changqing Chemical Group Co., Ltd., Changqing Oilfield Company, CNPC, Xi’an 710018, China; xdx2_cq@petrochina.com.cn (D.X.); guojian_cq@petrochina.com.cn (J.G.); lyubo_cq@petrochina.com.cn (Y.L.); 2 Shaanxi Province Key Laboratory of Environmental Pollution Control and Reservoir Protection Technology of Oilfields, Xi’an Petroleum University, Xi’an 710065, China; 23212071099@stumail.xsyu.edu.cn (D.X.); 24212071147@stumail.xsyu.edu.cn (J.J.); 3 Engineering Research Center of Oil and Gas Field Chemistry, Universities of Shaanxi Provence, Xi’an Petroleum University, Xi’an 710065, China; znpeng1_cq@petrochina.com.cn

**Keywords:** deep gas reservoir, reservoir repair, surfactants, condition-dependent remediation, multi-mechanism surfactant systems

## Abstract

Deep natural gas reservoirs are characterized by high temperatures, high pressure, high salinity, high condensate oil, and low permeability. Deep gas reservoirs frequently experience damage due to solid-skeleton destabilization, wellbore liquid loading and microscopic capillary liquid lock. These damages may occur independently or together under reservoir conditions. These damage modes may cooccur or interact in some deep gas reservoirs, and such scenarios can make single-function surfactant remediation insufficient. This review focuses on three functional classes of surfactants—clay stabilizers, foaming agents, and liquid-lock release agents—that target these three damage stages. Recent advances in molecular design, formulation optimization, and performance evaluation are summarized. Anionic, cationic, amphoteric, nonionic, as well as fluorocarbon-based surfactants whose headgroups may be ionic or nonionic each show distinct merits under given test conditions, yet most existing systems still experience limited salt/oil tolerance, weak adsorption stability, insufficient wettability reversal, and poor compatibility under extreme reservoir conditions. More importantly, current studies have largely focused on individual agents, while integrated remediation strategies that simultaneously stabilize the solid framework, enhance macroscopic liquid flow, and release microscopic capillary liquids are immature. Key bottlenecks include insufficient targeting in molecular design, unclear synergistic mechanisms, limited dynamic evaluation methods, and conflicts between environmental sustainability and cost. We propose that future research should prioritize obtaining a mechanistic understanding of interfacial synergies, static–dynamic coupled evaluation, and green low-cost synthesis, thereby accelerating the development of integrated reservoir repair agents for efficient and stable deep natural gas production.

## 1. Introduction

Under the guidance of China’s national goals for carbon peaking and carbon neutrality, energy structure transformation has become essential. As a clean and efficient low-carbon energy source, natural gas has significantly higher combustion efficiency than coal and oil, with carbon emissions per unit of heat value amounting to only 56% of that of coal and 71% of that of oil. Its applications in power generation, industrial fuel, and urban gas are expanding rapidly. According to data from the BP Statistical Review of World Energy, global natural gas consumption accounted for 24.3% of total primary energy consumption in 2023, and this proportion is projected to rise above 27% by 2030, making it a key pillar in the global energy transition [1]. However, as shallow and medium-depth gas reservoirs gradually enter the mid-to-late stages of development, the main frontiers of natural gas exploration and production have accelerated toward deep, ultra-deep, and low-permeability tight reservoirs. Although deep reservoirs in regions such as the Sichuan Basin, Tarim Basin, and Ordos Basin hold substantial resource potential, they commonly pose compounded challenges due to extreme geological conditions—including high temperatures (above 150 °C), high pressure, high salinity (total mineralization exceeding 5 × 10^4^ mg/L), high condensate content, and low permeability. Rapid declines in well productivity and difficulties in maintaining stable output after commissioning have become critical bottlenecks restricting efficient development [2]. The root cause of declining productivity in deep gas wells lies in the multi-stage, condition-dependent evolution of reservoir damage. During drilling, completion, and fracturing operations, work fluids invade the formation, triggering hydration and swelling of clay minerals, which leads to near-wellbore blockage by solid particles. During production, as formation pressure declines, accumulated liquids in the wellbore rise and create backpressure, resulting in macroscopic liquid-phase blockage. Even after liquid removal, capillary self-adsorption can still trap water or condensate phases within micro-pores and throats, causing water lock or hydrocarbon lock damage that severely reduces gas relative permeability. These three damage types are inter-related and may couple under reservoir conditions. They can independently initiate or interact with one another, generating multi-scale blockage effects across the matrix framework and pore-throat fluid pathways at both macroscopic and microscopic scales. Single-type chemical agents are insufficient to mitigate such coupled multi-scale formation damage. Surfactants, due to their unique amphiphilic molecular structure, can significantly alter the interfacial properties between solid-liquid and gas–liquid systems, demonstrating irreplaceable advantages in suppressing clay swelling, foam-based water drainage, and relieving liquid lock. They have thus become a core component of chemical reservoir restoration systems.

In recent years, scholars both domestically and internationally have conducted extensive research on three key chemical agents—clay stabilizers, foaming agents, and liquid-lock release agents—focusing on molecular structure design, formulation optimization, and performance evaluation methods, achieving a series of significant advances. Anionic, cationic, amphoteric, nonionic, and fluorocarbon surfactants each possess unique characteristics; however, single-function chemical agents may be insufficient when these damage modes are strongly coupled. Otherwise, individual or sequential treatments may remain adequate multifaceted performance requirements in harsh environments. Crucially, current research has primarily focused on enhancing the performance of individual functional agents, while integrated remediation agents targeting all three damage mechanisms simultaneously are still in their early stages of development. Systematic solutions addressing the entire chain—from solid-phase framework stabilization to macroscopic fluid flow guidance and microscopic capillary release—are lacking.

This review addresses three questions: (1) how clay-related matrix damage, wellbore liquid loading and pore-scale liquid lock occur and may couple in deep gas reservoirs; (2) what clay stabilizers, foaming agents and liquid-lock release agents can and cannot achieve under high-temperature, high-salinity and high-condensate conditions; and (3) what an integrated or staged treatment perspective adds beyond existing single-agent reviews. Reviews have mostly focused on one surfactant class or one damage type [3,4,5], and they have rarely linked the three damage stages, compared the condition-dependent performance limits of the three surfactant classes, or discussed the compatibility and injectivity constraints of combined formulations. Based on the condition-dependent evolution mechanism of reservoir damage in deep, complex gas reservoirs, this review therefore provides a cross-stage framework linking “solid-skeleton destabilization—macroscopic liquid loading—microscopic capillary liquid lock”. We systematically review the current research status and technological progress of clay stabilizers, foaming agents and liquid-lock release agents; critically analyze the core shortcomings of existing agent systems in terms of temperature, salinity and oil resistance, adsorption stability, synergistic mechanisms and evaluation methods; and distinguish when an integrated formulation is preferable—when multiple damage types spatially overlap and compatibility/injectivity have been verified—from when sequential or staged treatments are more appropriate. It further outlines future directions for integrated reservoir repair agents from four dimensions: targeted molecular design, elucidation of formulation synergy, development of dynamic evaluation systems, and green, low-cost synthesis processes, aiming to provide theoretical insights and technical references for the efficient exploitation of deep, complex gas reservoirs.

## 2. Mechanism of Decline in Gas Well Production Capacity 

**Figure 1:**
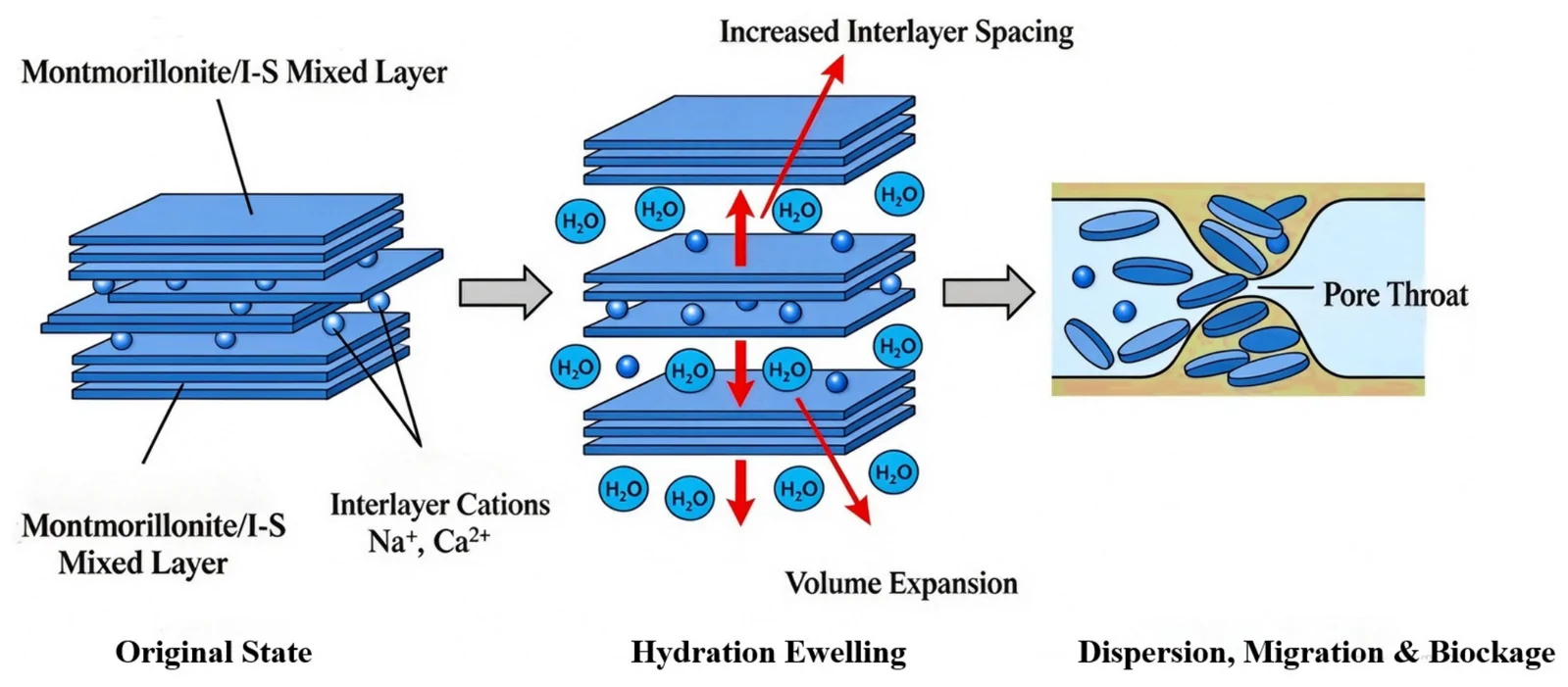
Diagram of the Mechanism of clay hydration expansion. The red arrows indicate the increase in interlayer spacing and the resulting volume expansion of the clay layers.

During the entire life cycle of drilling and completion, fracturing, and production of gas wells, reservoir damage is the main factor restricting production capacity. In the drilling and completion, fracturing, and production life cycle of low-permeability sandstone and tight condensate gas reservoirs in Longqing Oilfield, the decline in gas well production capacity has not been caused by a single factor, but rather has involved multiple damage modes to the matrix, wellbore, and pore throats. Their locations, mechanisms, and possible interactions are summarized below; the sequence is not universal and depends on reservoir and production conditions (Figure 1):

(1) Stage 1: Matrix solid-particle blockage near wellbore

Deep gas reservoirs often contain relatively high contents of clay minerals such as montmorillonite and illite. When external working fluids (e.g., drilling fluid and fracturing fluid) invade the reservoir, water molecules penetrate the interlayer gaps of clay minerals, triggering hydration and ion exchange with interlayer cations such as Na^+^ and Ca^2+^. This increases the crystal -layer spacing and causes volume expansion, followed by dispersion and migration of clay particles, which block pore throats and sharply reduce permeability [6,7,8,9,10,11,12,13]. Permeability losses caused by clay swelling have been reported as 30–80%, making this one of the most significant damage types in deep gas reservoirs. This solid-particle blockage directly damages the porous medium framework and is the first damage type considered in this review. It should be noted, however, that its severity depends on clay type, content, fluid salinity, and confining conditions, and it may occur independent of the later-described wellbore and pore-scale damage.

(2) Stage 2: Macroscopic liquid loading in the wellbore

During condensate–gas–reservoir development, formation pressure decline causes condensate oil and formation water to accumulate at the bottom hole and within the wellbore, forming a liquid column that raises bottom-hole backpressure and sharply reduces gas productivity [14,15]. In China, more than 40% of the producing gas wells in large condensate gas fields such as Sulige and Daniudi have experienced productivity loss exceeding 30% due to liquid loading, and some wells have been shut in by “water flooding” [16,17]. Conventional foam-drainage agents face two coupled problems under such conditions: (i) high formation-water salinity (total mineralization can exceed 5 × 10^4^ mg/L) destabilizes the foam film [18], and (ii) condensate oil (5–30%) spreads at the gas–liquid interface and accelerates foam rupture, making defoaming difficult [19,20,21,22]. These effects depend on salinity, condensate content, temperature, and foam-agent structure, and they are not necessarily preceded by the clay-related damage described in Stage 1.

(3) Stage 3: Deep micro-level liquid lock sealing

**Figure 2:**
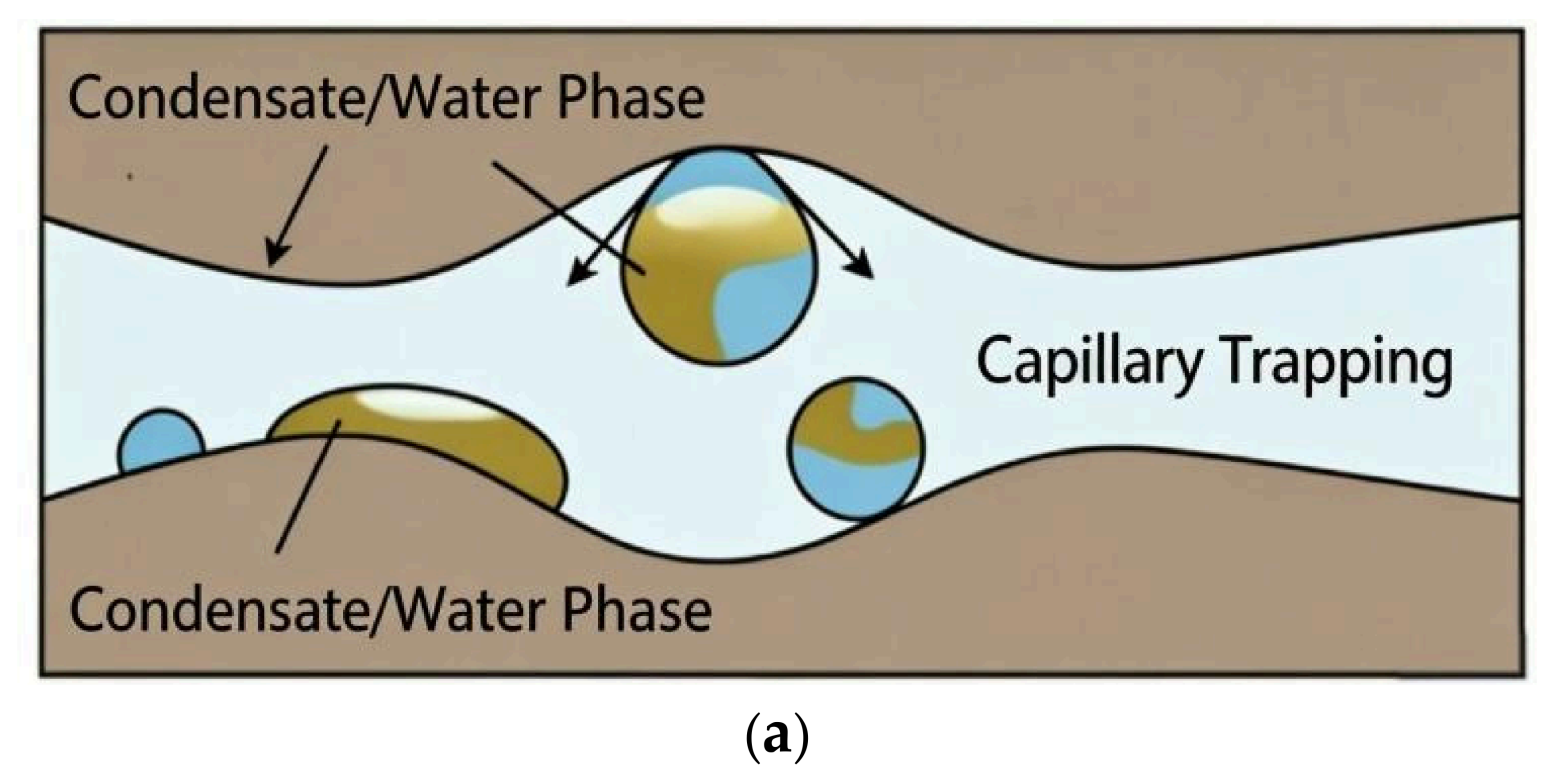

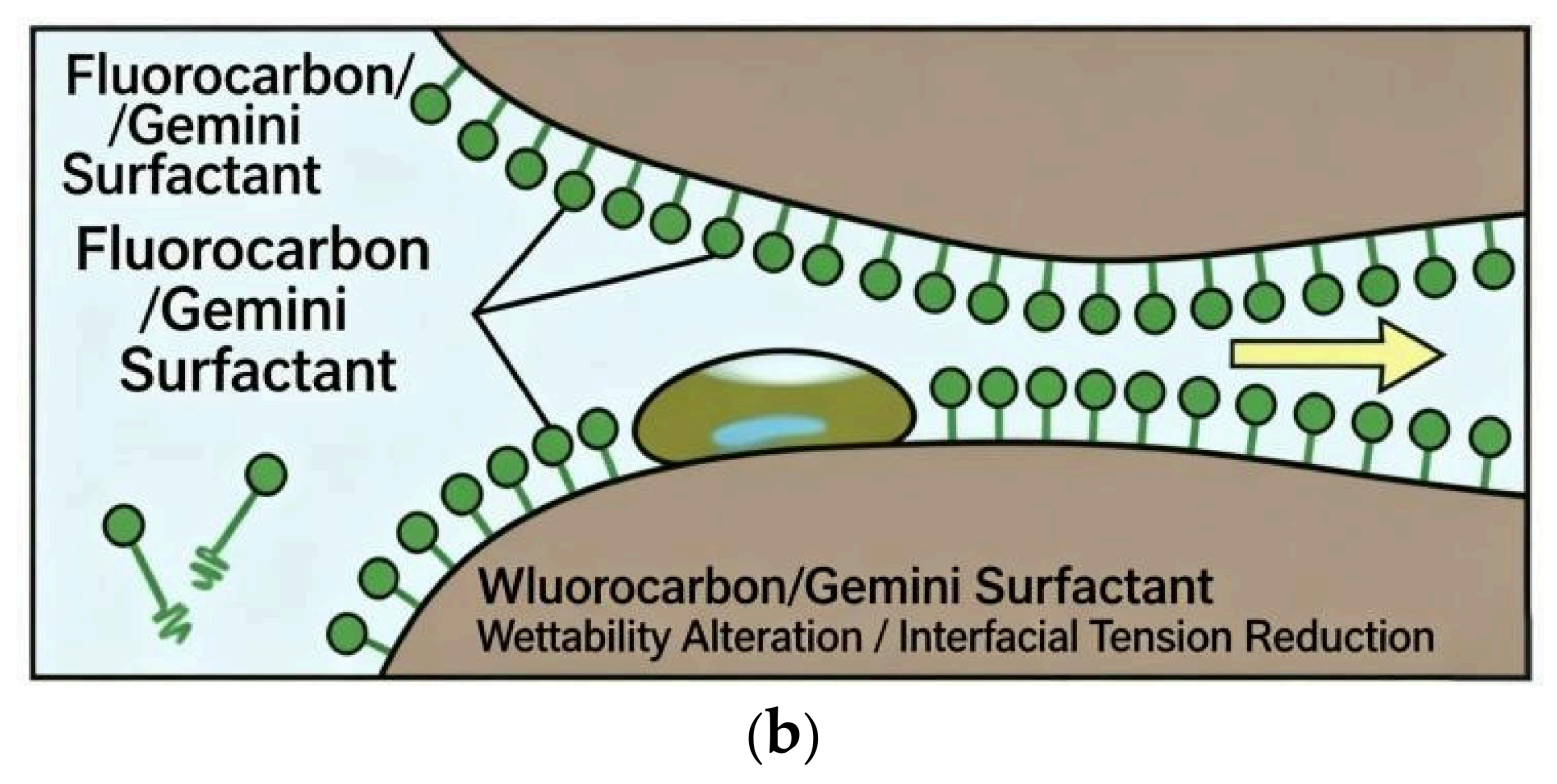
Liquid lock damage and wetting reversal. (**a**) Capillary trapping of condensate by capillary force; the arrows show the flow direction of trapped condensate. (**b**) Wettability alteration and interfacial tension reduction after adding fluorocarbon/Gemini surfactant; the arrow indicates the flow path of fluid after liquid lock removal.

Pore-scale liquid trapping can occur independent of wellbore liquid loading, or persist after wellbore liquid unloading, depending on capillary pressure, wettability, and condensate distribution. In ultra-deep or low-permeability gas reservoirs, water or condensate can form water locks or hydrocarbon locks through capillary self-absorption and retention [23,24] (Figure 2a). This reverse suction effect can lead to a significant reduction in the relative permeability of the gas phase [25], and, in severe cases, even when the pressure difference reaches over 25 MPa, the gas well cannot be restarted. In ultra-deep condensate gas reservoirs and low-permeability gas reservoirs [26], the accumulated liquid in the wellbore is prone to form water locks or hydrocarbon locks through capillary self-suction, resulting in a significant reduction in the relative permeability of the gas phase. Traditional liquid lock removal agents mostly rely on anionic surfactants, which have poor temperature and salt resistance and limited wetting reversal effects, so they do not adapt to harsh formation conditions [27,28] (Figure 2b). 

**Figure 3:**
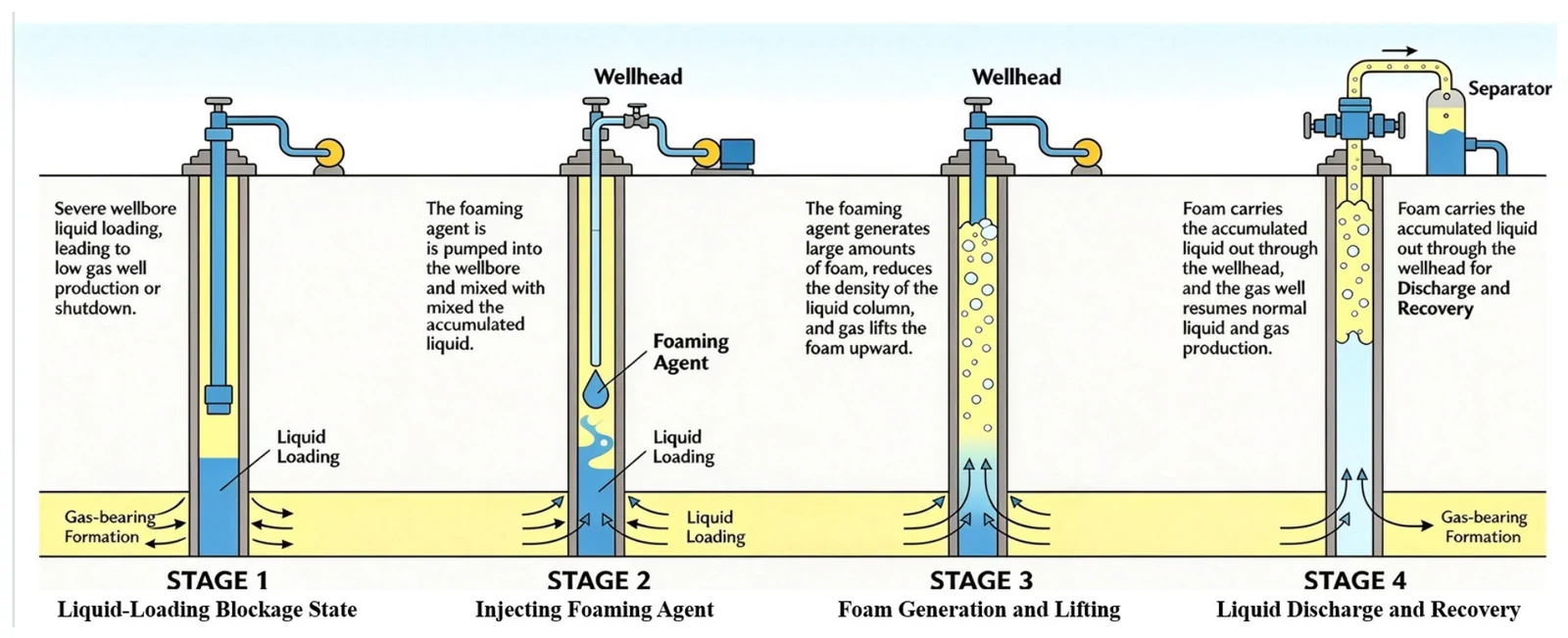
Wellbore liquid accumulation and foam drainage gas recovery. Arrows indicate the flow direction of gas and liquid in the wellbore and formation.

In summary, the three damage modes discussed above differ in occurrence location and mechanism—near-wellbore matrix, wellbore, and micro-pore throats. They may occur independently or co-occur under certain reservoir and production conditions, but the literature has not established a universal temporal sequence. Figure 3 illustrates possible co-occurrence and interaction pathways rather than a mandatory treatment order [29].

## 3. Application of Surfactants in Natural Gas Development

Surfactants, as a class of chemical substances with a special amphiphilic molecular structure (hydrophilic group and hydrophobic group), can significantly alter the properties of material interfaces. They have unique advantages in reducing surface/interface tension, regulating wettability, and stabilizing foams, and have become key, widely used chemical agents in gas reservoir development [30].

### 3.1. Functions of and Issues with Surfactants for Gas Field Production

(1) Anionic Surfactants

When these chemicals are dissolved in water, their active components carry negative charges. Common examples include fatty alcohol polyoxyethylene ether sulfates (AESs), carboxylates, and sulfonates [31]. Certain anionic surfactants can exhibit good thermal stability and foaming ability under the reported test conditions; their tolerance to high-valence cations is limited. Since the surfaces of most gas reservoir sandstones carry negative charges, there is electrostatic repulsion between anionic surfactants and rocks, resulting in relatively low adsorption loss in the formation [32]. High-valent metal ions such as calcium and magnesium have weak tolerance and are prone to precipitation in high salinity brine [33].

(2) Cationic surfactants

**Figure 4:**
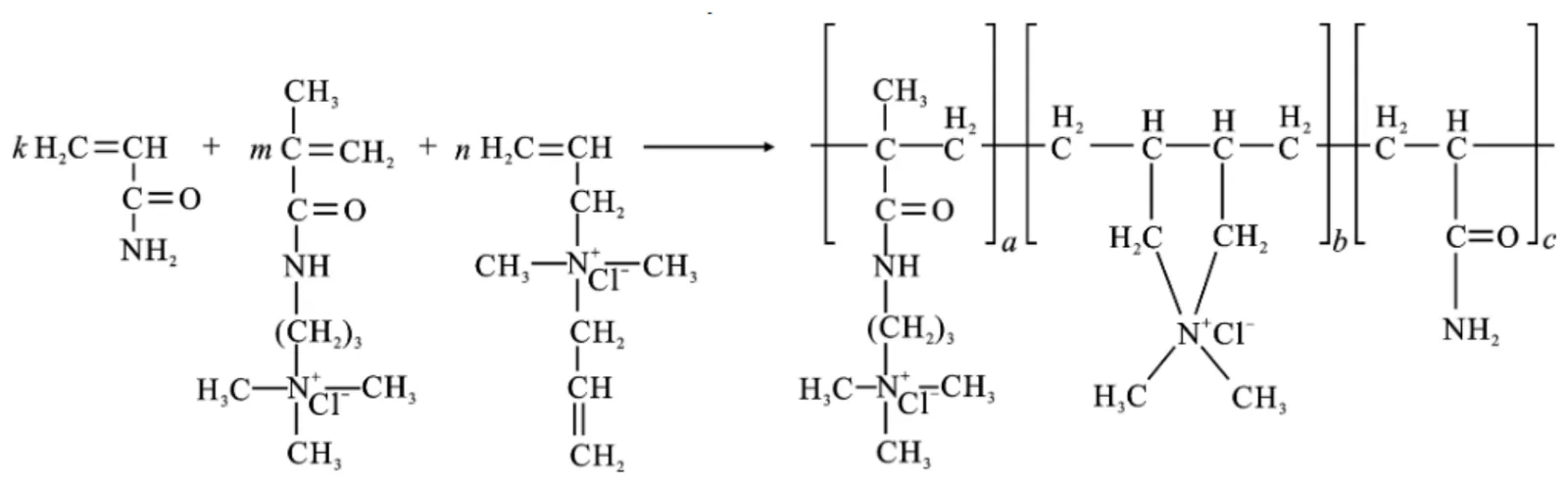
Reaction equation of PDMD.

Their active components carry positive charges and mainly include quaternary ammonium salts and heterocyclic compounds. Certain cationic surfactants show strong biocidal and clay-swelling-inhibiting effects, depending on headgroup, chain length, counterion, concentration, temperature, and salinity. They have huge adsorption capacity in sandstone formations and often undergo compatibility reactions with anionic agents to form precipitates. Currently, similar cationic polymers such as the PDMD synthesized by Zhang [34] (Figure 4) have relatively large molecular weights and are relatively difficult to synthesize, which limit their large-scale application [35].

(3) Amphoteric surfactants

**Figure 5:**
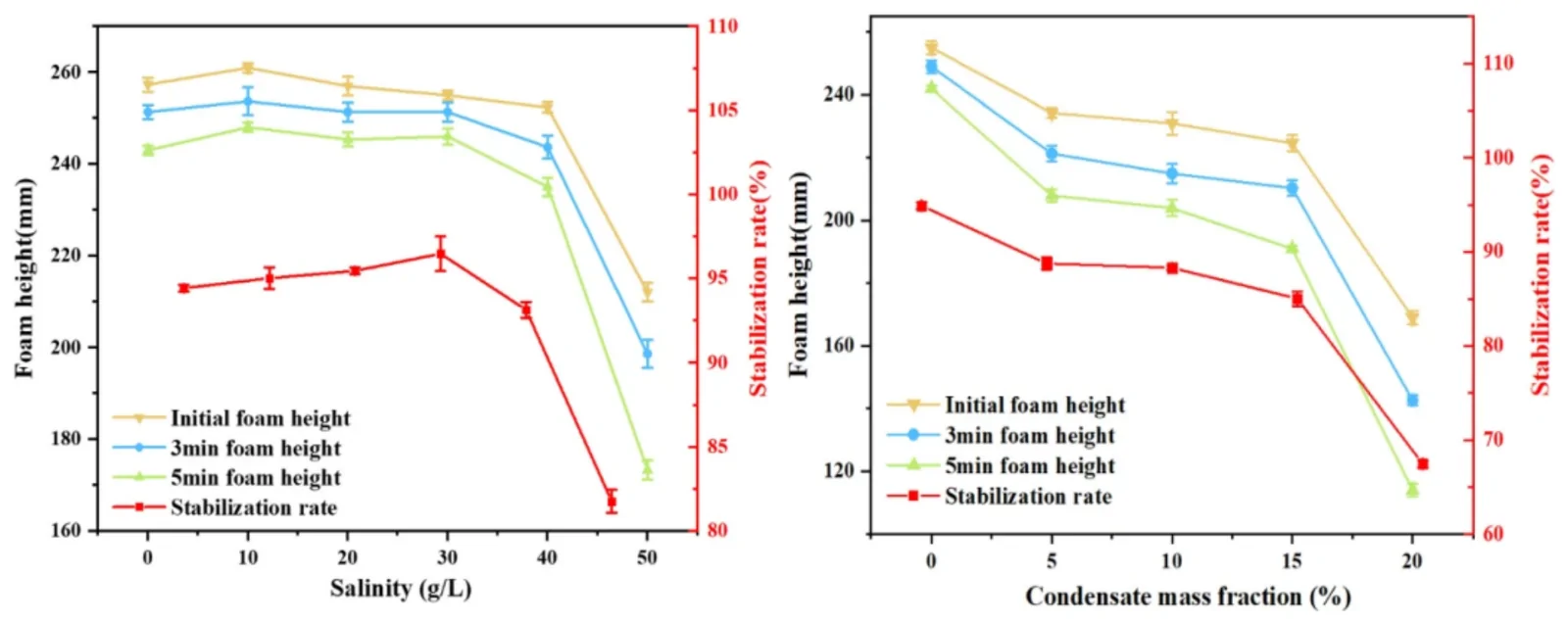
Influence of mineralization degree and condensate oil on foaming performance of XY-1 foam agent.

These surfactants have both negative and positive polar groups in their molecular structure. Their electrical properties change with the pH value of the medium. Typical examples include the betaine type and imidazoline type. They can show good hard-water and salt tolerance under certain conditions, but performance depends on molecular structure, electrolyte concentration, and temperature. The synthesis process is relatively complex, and the raw material cost is high. They are used as the main agent or additive in high-performance composite systems [36]. synthesized a sulfonic acid betaine surfactant XY-1 as a foam drainage agent (foaming agent) [37], which has certain salt and oil resistance capabilities, but its performance is insufficient (Figure 5).

(4) Non-ionic surfactants

These surfactants do not ionize in aqueous solutions. They achieve solubility by forming hydrogen bonds with water molecules through their polar functional groups (such as polyoxyethylene chains), and their stability is extremely high, being almost unaffected by fluctuations in formation mineralization or pH value [38]. Their critical micelle concentration (CMC) is usually low, meaning that excellent surface tension reduction effects can be achieved at relatively low concentrations. However, there is a “cloud point” phenomenon, where phase separation occurs when the temperature rises to a certain value. Therefore, specific modifications are required for applications in ultra-deep and ultra-high-temperature gas reservoirs. Although these surfactants have been widely used, they still face various challenges in extreme gas reservoir environments.

In the field of clay stabilization, cationic surfactants can neutralize the negative charges on the surfaces of clay particles through electrostatic adsorption, compress the double electric layer, and inhibit hydration expansion [39]. In the field of foam drainage, amphoteric surfactants can form a dense and tough interface at the gas–liquid interface, enhancing foam stability. In the field of desorption lock, fluorocarbon surfactants have excellent hydrophobic and oleophobic properties and can weaken capillary force through the wetting reversal mechanism to promote the return flow of retained liquid phase [40].

However, traditional surfactants still have significant shortcomings in the complex conditions of deep gas reservoirs: cationic surfactants are prone to thermal decomposition in high-temperature and high-mineralization environments and have insufficient adsorption stability; anionic surfactants have poor salt resistance and are prone to forming precipitates with Ca^2+^ and Mg^2+^ in formation water; single-type surfactants have a single function and cannot meet multiple performance requirements. Developing new structural surfactants and optimizing compound systems have become the core directions to break through the existing technical bottlenecks [41].

### 3.2. Research Progress on Clay Stabilizers

(1) Traditional Clay Stabilizers

**Figure 6:**
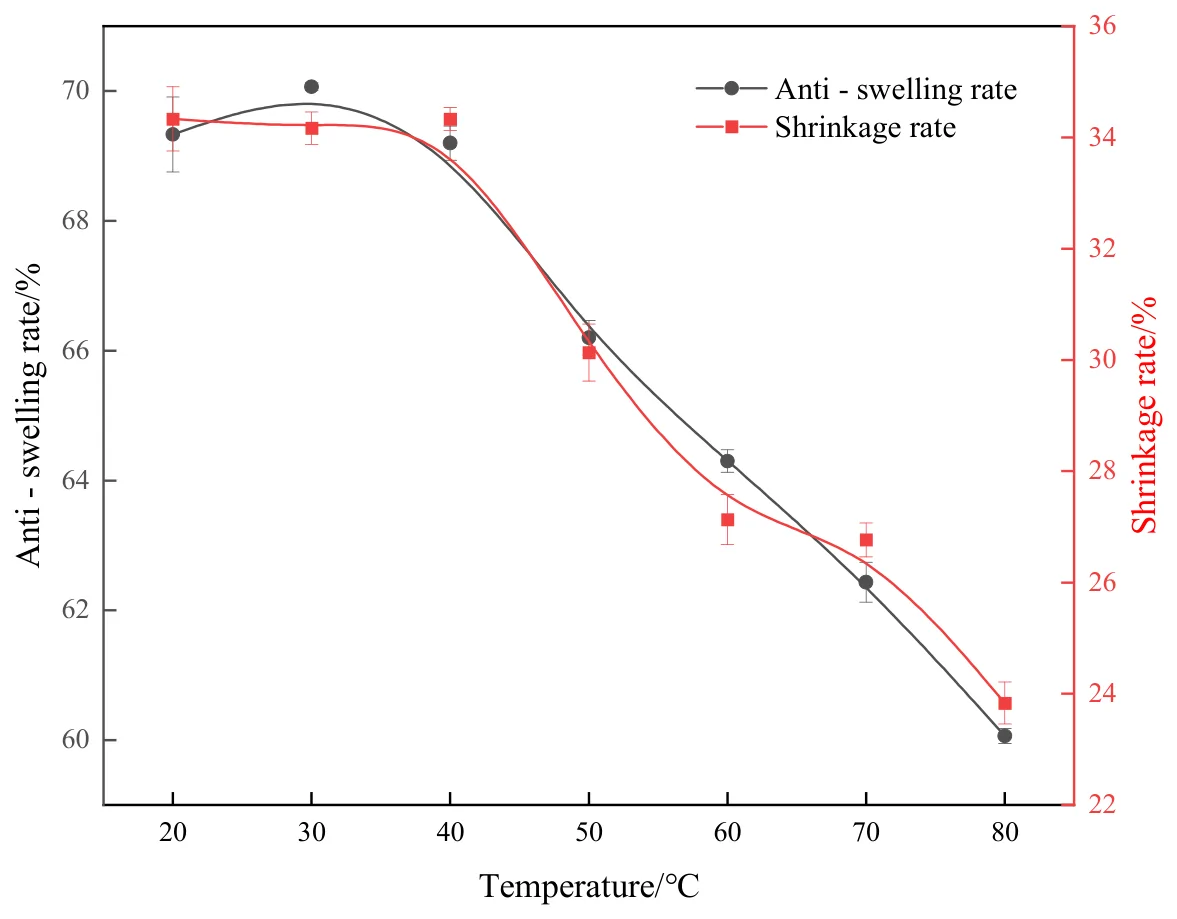
Graph showing variations in anti-expansion rate and shrinkage rate with temperature.

The clay stabilizers developed domestically and internationally mainly include inorganic salts, polymers, and cationic surfactants. Inorganic salts (such as KCl, NH4Cl) displace Na^+^ in the clay layer through ion exchange, which is cost-effective but has limited anti-expansion effects and poor water-washability and is prone to loss with formation water, making it difficult to achieve long-term stability. Polymer-based ones (such as polyquaternium) form a protective film on the surface of the clay through molecular chain adsorption, providing a long-lasting anti-expansion effect, but they have large molecular weights and are prone to clogging of reservoir pore throat channels, so are especially not suitable for low-permeability gas reservoirs. As shown in Figure 6, traditional cationic surfactants (such as dodecyltrimethylammonium chloride) have strong adsorption capacity, but in high-temperature and high-mineralization environments, there are problems such as insufficient adsorption capacity and poor stability of the hydrophobic membrane, and their inhibitory performance is prone to attenuation.

(2) New cationic surfactants

**Figure 7:**
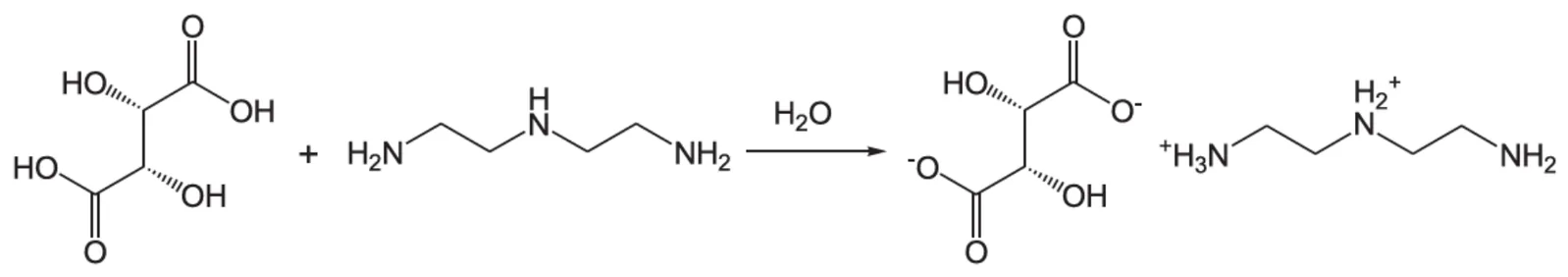
Synthesis of amine-tartarate inhibitor.

Gemini cationic surfactants, due to their unique structure of dual hydrophilic groups and dual hydrophobic groups, exhibit significant advantages in interface adsorption ability and anti-expansion performance, becoming a research hotspot in recent years [42]. Al-Amiery et al,2023 [42] synthesized Gemini surfactants with a Mannich base structure, achieving an anti-expansion rate of over 85% at 100 °C, but the mineralization resistance was only 1 × 10^4^ mg/L. Chen et al,2017 [43] developed amine-tartarate type clay stabilizers, with excellent anti-expansion effect, but the synthesis process was complex, and the cost was high (Figure 7).

Domestically, Feng et al,2019 [44] prepared cationic hyperbranched polymer clay stabilizers. At 120 °C and a mineralization degree of 2 × 10^4^ mg/L, the anti-swelling rate remained around 90%, but the molecular chains were prone to remaining in the pores of low-permeability reservoirs. Synthesized low-molecular-weight quaternary ammonium salt polymers, which balanced the anti-swelling performance and injectability. However, the water-wash resistance still needs to be improved. Studies have shown that by introducing functional groups such as hydroxyl and ether bonds into the molecular structure, the hydrogen bond interaction between the surfactant and the clay particles can be enhanced, improving the adsorption stability. However, studies on adaptability to ultra-high temperature (above 150 °C) and ultra-high mineralization (above 5 × 10^4^ mg/L) conditions in deep gas reservoirs are still relatively lacking.

### 3.3. Research Progress on Foaming Agents for Foam Drainage

(1) Shortcomings of Traditional Foaming Agents

The core features of foaming agents includes foaming ability, foam stability, salt tolerance, and oil tolerance. Traditional foaming agents mainly consist of anionic surfactants (such as SDS, AES) and non-ionic surfactants (such as the AEO series). Anionic surfactants have strong foaming ability and the double electric layer is compressed in high-salinity environments, making the foam liquid film prone to instability; non-ionic surfactants have better salt tolerance but weak foaming ability and short foam half-life. Dong et al, 2026 [45] synthesized a cocoyl amino-acid-based Gemini surfactant. The dual-hydrophilic/dual-hydrophobic structure was introduced to enhance interfacial activity. This Gemini surfactant showed favorable foam volume and half-life under the tested conditions, but the synthesis process is complex and difficult to control.

**Figure 8:**
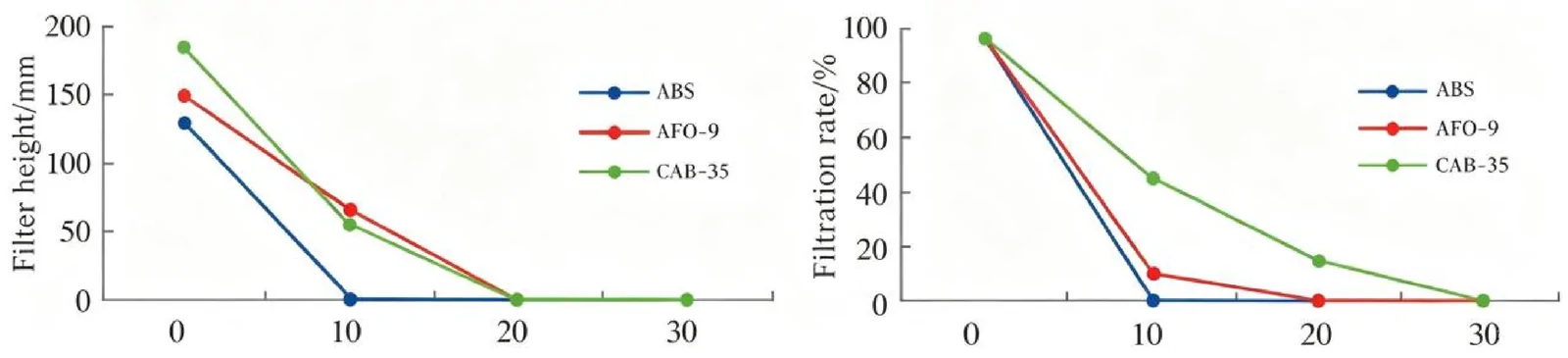
Relationship between foaming performance and condensate oil concentration.

In recent years, zwitterionic surfactants have attracted wide attention for foam-drainage applications, because they combine some favorable properties of cationic and anionic surfactants under certain test conditions. Their performance still depends strongly on salinity, condensate-oil content and temperature. Sodium lauryl amine propyl betaine (CAB) and lauryl amine propyl hydroxysulfonate betaine (LABS) have been applied in the field. However, in environments with high condensate oil content (above 20%), the oil phase tends to spread on the gas–liquid interface, replacing the adsorption sites of the foam drainage agent, resulting in rapid foam rupture. Xiong et al, 2025 [46]. reported that traditional zwitterionic surfactants show a marked loss of foam stability when the condensate-oil content exceeds 25%, making it difficult to meet the drainage requirements (Figure 8) [47]. Because Figure 8 reports foam height/filtration rate rather than a time-resolved foam-decay curve, we do not extract a specific half-life value from this figure.

(2) Salt-tolerant and oil-resistant foam agents

**Figure 9:**
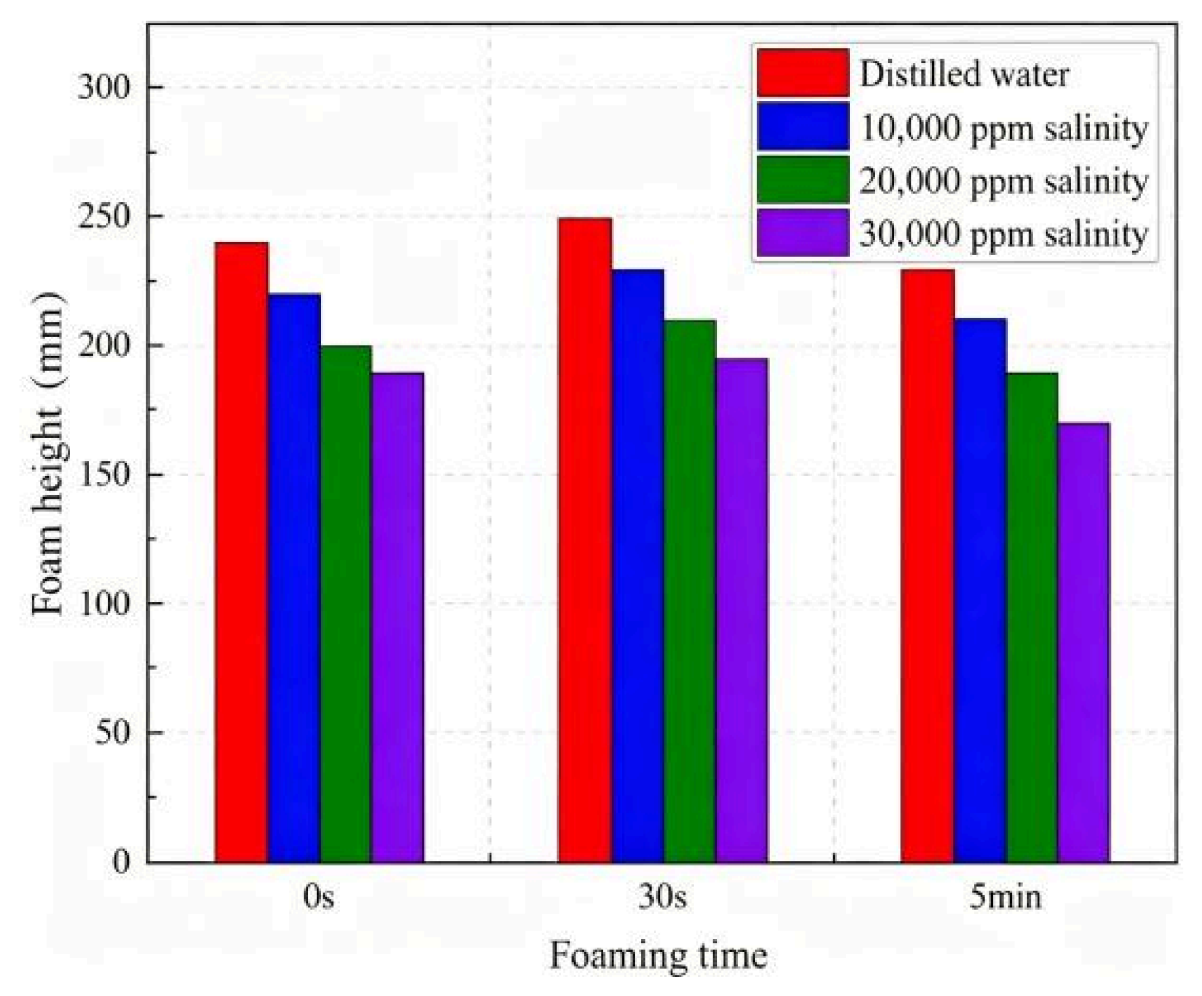
Effect of salinity on foaming performance [47].

To enhance the performance of foam agents under complex conditions, researchers have mainly carried out their work in two directions: one is molecular structure modification, introducing long-chain alkanes, sulfonates and other functional groups into the surface-active agent molecules of the betaine type to enhance the interfacial adsorption ability and anti-oil interference ability; the other is the optimization of the compound system, by combining anions with amphoteric ion surface-active agents to utilize the synergistic effect to improve the foam stability. Fan et al, 2025 [48] completed the compound formulation, and the salinity has a significant impact on the foaming property, as shown in Figure 9.

Internationally, researchers have synthesized fluorinated betaine surfactants. Under a mineralization degree of 5 × 10^4^ mg/L and a condensate oil content of 30%, the foam half-life exceeded 60 min [47,49]. However, the fluorinated compounds caused significant pollution and did not easily degrade, failing to meet environmental protection requirements. Mohanty et al. [50] developed a system combining AES with coconut alkyl amide betaine, which enhanced the salt tolerance to 3 × 10^4^ mg/L, but the performance in resisting condensate oil still needed improvement.

**Figure 10:**
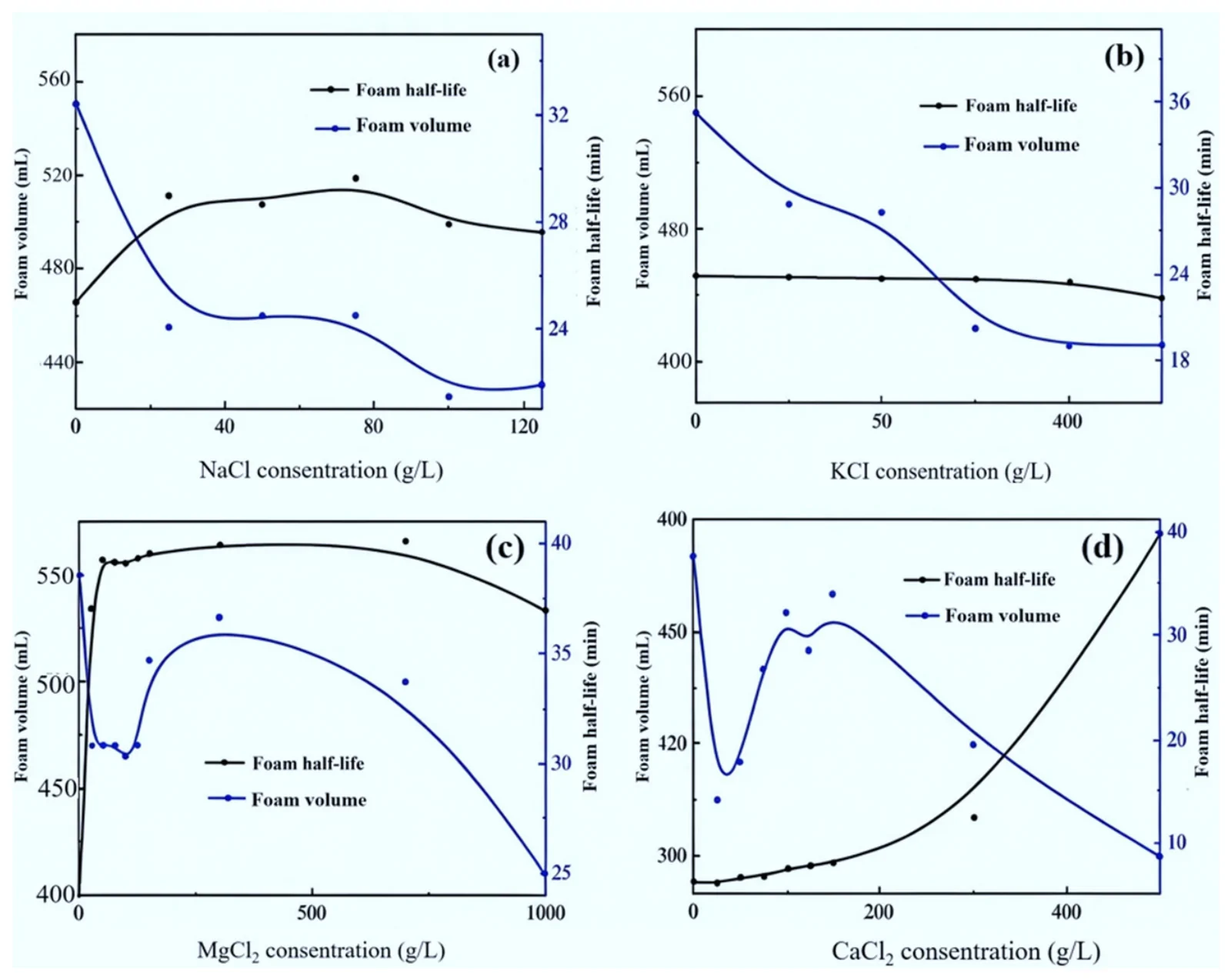
Effects of salt concentration on foam volume and foam half-life of the composite foam drainage agent at 30 °C: (**a**) NaCl, (**b**) KCl, (**c**) MgCl_2_, (**d**) CaCl_2_. Data adapted from Ref. [51].

Dong et al. developed a composite surfactant foam agent [51]. The data in Table 1 were obtained at 30 °C with condensate oil as the oil phase and were adapted from Ref. [52] Tables 4–8); they are not from the methanol tests reported in Ref. [51]. As shown in Table 1, foam height decreased with time under all tested condensate-oil concentrations, and the decrease became more pronounced at higher condensate-oil concentrations. Figure 10 [51] separately shows the effects of NaCl, KCl, MgCl_2_ and CaCl_2_ concentration on foam volume and half-life: foam volume decreases with increasing NaCl and KCl concentration, whereas it increases with increasing MgCl_2_ and CaCl_2_ concentration; in the CaCl_2_ system, the foam half-life also increases with CaCl_2_ concentration under the tested conditions. Therefore, the effect of salinity on foam performance is ion-specific and cannot be generalized as a monotonic decline, whereas increasing condensate-oil content consistently accelerates foam decay. Improved the temperature resistance of a modified tartrate surfactant by introducing polyether segments, reaching up to 140 °C [53]. However, the synthesis cost is high, limiting its large-scale application. Current research still faces a core challenge: achieving the coordinated improvement of salt resistance, oil resistance, and long-term stable foaming performance of a foam agent while balancing environmental friendliness and economic efficiency [54].

### 3.4. Research Progress on Liquid-Lock Release Agents

It should be noted that the term ‘surfactants’ in this review strictly refers to amphiphilic molecules with distinct hydrophilic and hydrophobic groups. Inorganic salts (e.g., KCl, NH_4_Cl) and alcohol cosolvents (e.g., methanol, ethylene glycol) are not classified as surfactants. They are discussed in this section only as additives or comparative agents, acting through ion exchange or interfacial co-solvent effects. In this review, “liquid-lock release agents” refers to surfactants that reduce gas–liquid interfacial tension, alter rock wettability, weaken capillary force, and promote flowback of trapped water or condensate from micro-pores and throats. The term does not imply removal of silica or “desilication”.

(1) Mechanisms and Types of Liquid-Lock Release Agents

**Figure 11:**
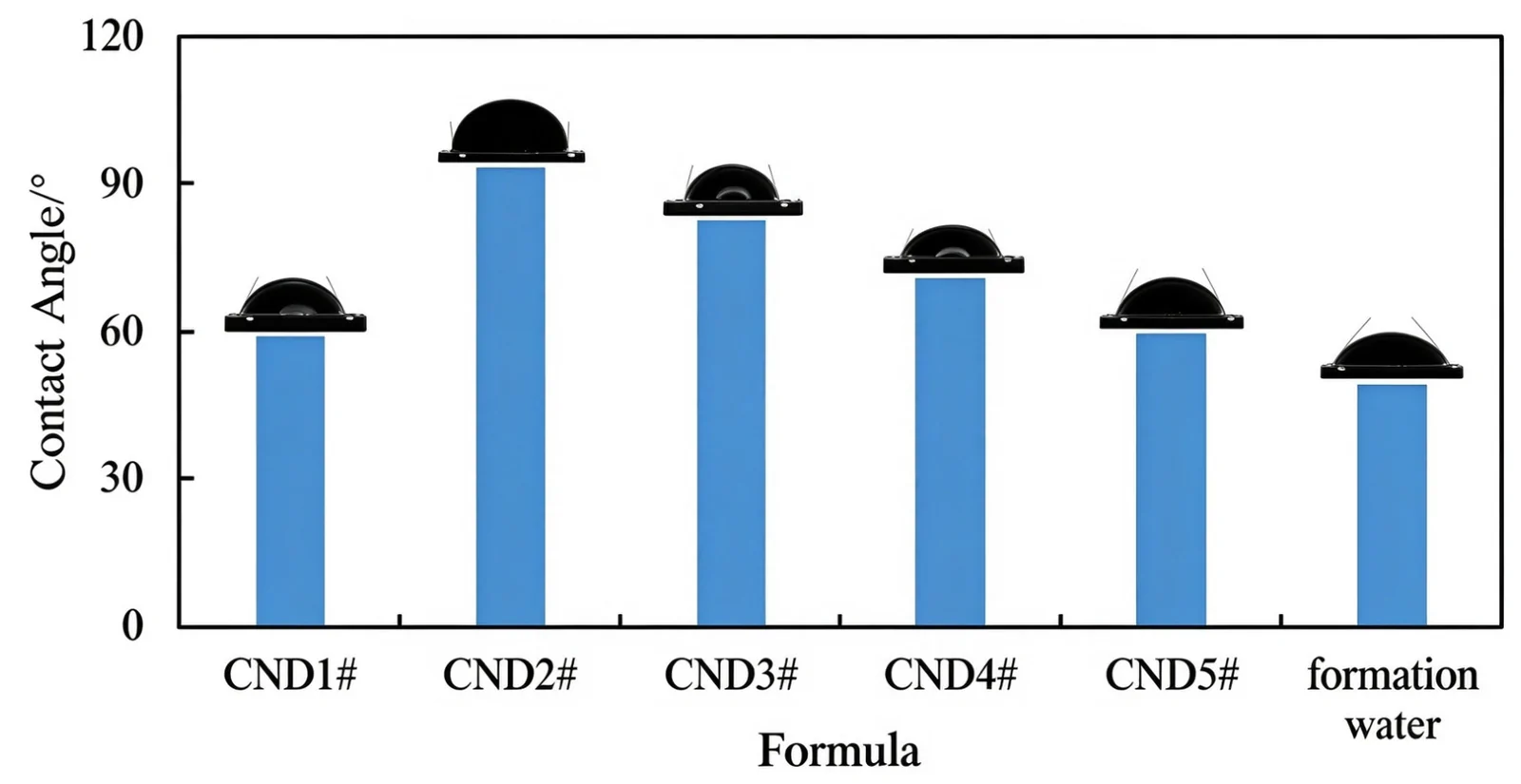
Contact angle of core after immersion with nano treatment agent.

Liquid-lock release agents work by reducing the interfacial tension between gas and liquid, altering the wetting property of rocks, weakening the capillary force, and promoting the return flow of trapped liquid phase. The core mechanisms include reduced interfacial tension and wetting reversal. Traditional liquid-lock release agents mainly include anionic surfactants, cationic surfactants, and aliphatic compounds [55]. Anionic surfactants (such as sodium dodecylbenzenesulfonate) can effectively reduce interfacial tension, but they have poor salt tolerance and may fail in high-salinity environments; cationic surfactants (such as cetyltrimethylammonium bromide) can achieve wetting reversal of rocks, but their adsorption stability is insufficient, and they are prone to desorption; aliphatic compounds (such as methanol, ethylene glycol) can reduce the viscosity of the system and assist in the return flow of the liquid phase, but their liquid-lock release performance is limited, and they are prone to causing secondary damage. In China, as shown in Figure 11, synthesized long-chain alkyl oxamide surfactants, which achieved a core-periphery permeability recovery rate of over 85% under a salinity of 3 × 10^4^ mg/L [56], but the contact angle was not satisfactory. improved the temperature resistance to 150 °C by compounding oxamide with non-ionic surfactants, but the liquid-lock release performance significantly decreased in the presence of condensate oil. Reported liquid-lock release agents often lack dynamic core-flooding data, condensate tolerance, and long-term adsorption stability.

(2) Research Progress on New Liquid-Lock Releasing Agents

In recent years, new liquid-lock releasing agents such as fluorinated surfactants have become a research hotspot. Under specific laboratory evaluation conditions, fluorocarbon surfactants can deliver high chemical stability, broad temperature-salinity adaptability and low interfacial tension. Nevertheless, such outstanding performance cannot be guaranteed in all reservoir environments.

**Figure 12:**
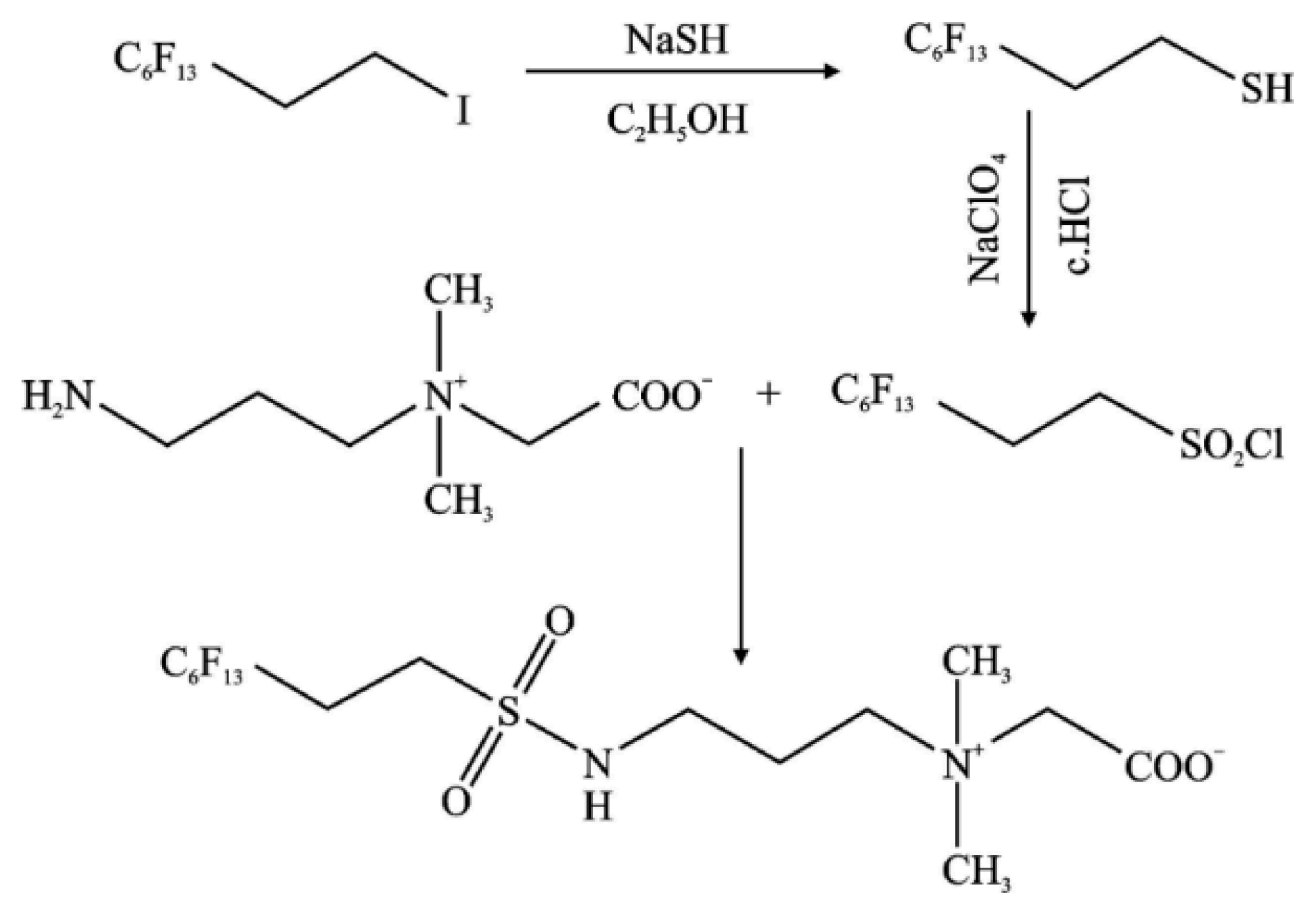
Synthesis Route of Fluorine-containing Solution Locking Agent.

The fluorine-containing groups in their molecules can form a dense adsorption film on the rock surface, achieving wettability reversal from liquid-wetting to gas-wetting (gas-philic) state. However, they struggle to simultaneously meet multiple performance requirements such as reduced interfacial tension, wetting reversal, and strong stability. Moreover, they are often synthesized in complex forms and require the combination of nanomaterials to change the roughness to release the liquid lock, as shown in Figure 12, and the microscopic mechanism of the liquid-locking release process has not been deeply studied [57].

It should be noted that the performance of individual clay stabilizers, foaming agents, and liquid-lock release agents cannot be directly extrapolated to mixed formulations, because competitive adsorption, electrostatic complexation, precipitation, and phase separation may substantially change the interfacial behavior and injectivity of the combined system.

## 4. Conclusions and Outlook

### 4.1. Limitations of Current Surfactant Systems

Taken together, the reviewed studies support several condition-dependent findings. First, clay hydration, swelling, and particle migration can cause significant permeability loss in water-sensitive deep gas reservoirs. Cationic surfactants/polymers, Gemini surfactants, and amine-tartrate inhibitors can reduce swelling under the tested conditions. Second, foam drainage can mitigate wellbore liquid loading, but foam stability is strongly affected by salinity, condensate oil, temperature, and test method. Third, liquid-lock release agents can reduce gas–liquid interfacial tension and/or alter rock wettability, and some laboratory systems improve contact angle or permeability recovery. These findings are summarized in Section 3.2, Section 3.3 and Section 3.4 and Table 2. Importantly, the reported performance is condition-specific and depends on concentration, temperature, pressure, brine composition, condensate content, and evaluation protocol.

First, molecular design remains insufficiently targeted. Most conventional surfactants adopt universal structures and cannot simultaneously tolerate extreme temperature, salinity, and condensate oil content. Clay stabilizers often face a trade-off between long-term anti-swelling performance and low-permeability injectability; foam drainage agents may show good initial foaming but suffer rapid film rupture under high salinity and condensate oil content; liquid-lock release agents frequently exhibit limited wettability reversal and poor adsorption stability. As a result, single-function agents cannot address the condition-dependent damage chain of “solid-skeleton destabilization—macroscopic liquid loading—microscopic capillary liquid lock.”

Second, formulation development still relies heavily on empirical trial and error. The adsorption competition, mixed micelle formation, interfacial packing, and intermolecular cooperative effects among different surfactant types at clay, gas–liquid, and rock interfaces have not been systematically clarified. Without a quantitative understanding of composition–performance relationships, it is difficult to precisely control component ratios and achieve synergistic improvements in multiple properties.

Third, the current evaluation systems are largely static and laboratory-based. Anti-swelling rate, foam half-life, and contact angle are useful basic indicators, but they cannot reproduce the dynamic flow, backpressure, capillary trapping, and condensate spreading that occur in real wellbores. The lack of core dynamic displacement, microfluidic visualization, and high-temperature/high-pressure dynamic testing has led to weak correlations between static screening results and field performance.

Fourth, there remains a contradiction between environmental friendliness and economic efficiency. Certain fluorinated surfactants can provide good temperature, salt, and oil tolerance under specific conditions, but their persistence, lack of degradation, and ecological risks limit large-scale application. Meanwhile, many environmentally acceptable modified surfactants require complex synthesis and expensive raw materials, restricting industrial promotion.

Fifth, an additional limitation concerns the integration of multiple functions into one treatment system. Although integrated formulations are conceptually attractive, their practical feasibility should not be overestimated. First, cationic clay stabilizers may complex or precipitate with anionic foaming or liquid-lock release agents, particularly in high-salinity and high-hardness brines. Second, competitive adsorption at clay and rock surfaces can deplete one active component and alter mixed interfacial packing; cationic species adsorb strongly on negatively charged sandstones, whereas nonionic or zwitterionic species may be displaced. Third, high-valence ions, condensate oil, and macromolecular or aggregated components may reduce solubility, destabilize foam films, or impair injectivity in low-permeability pore throats. Fourth, the three damage types occur in different locations—near-wellbore matrix, wellbore, and micro-pore throats—so a single combined slug may not contact all targets. Therefore, integrated formulations should be considered only when the damage zones spatially overlap and when compatibility and injectivity have been experimentally verified; otherwise, sequential or staged treatments may be more appropriate. Importantly, most reported composite systems provide only partial evidence, and dynamic high-temperature/high-pressure core-flooding data and field validation for true three-function integration remain scarce.

### 4.2. Future Research Directions

To overcome these bottlenecks, we propose that future research should explore condition-dependent integrated or staged remediation, while single-function optimization remains necessary. Four priority directions are proposed.

(1) Targeted molecular structure design

Molecular engineering should be guided by specific damage mechanisms and reservoir conditions. For clay stabilizers, functional groups such as hydroxyl, ether, sulfonate, and low-molecular-weight quaternary ammonium units can be introduced to enhance adsorption on clay surfaces, compress the electrical double layer, and reduce pore-throat blockage. For foam drainage agents, betaine-based amphoteric structures, anionic–amphoteric hybrids, and salt-tolerant hydrophobic tails should be designed to resist condensate spreading and improve foam film strength. For liquid-lock release agents, fluorine-free or short-chain degradable surfactants with strong wettability reversal, low interfacial tension, and high adsorption stability should be prioritized. A long-term research goal is the development of an agent that simultaneously resists high temperature, high salinity, and high condensate oil content.

(2) Systematic elucidation of synergistic mechanisms and integrated formulations

The interfacial behavior of mixed surfactant systems should be studied using molecular simulation, interfacial rheology, adsorption kinetics, and spectroscopic methods. Key issues include competitive adsorption, mixed monolayer formation, electrostatic and hydrophobic interactions, and the coupling between clay stabilization, foam stabilization, and wettability alteration. In particular, compatibility screening should be integrated into formulation design: cationic clay stabilizers may precipitate with anionic foaming or liquid-lock release agents; high-valence ions and condensate oil may destabilize mixed systems; strong adsorption of cationic species on negatively charged sandstone may deplete active components; and macromolecular or aggregated species may impair injectivity in low-permeability pore throats. Because the three damage types occur in different locations, a single combined slug may not contact all targets. Mechanism-guided decision criteria should therefore be established to define when an integrated formulation is preferable—for example, when multiple damage types spatially overlap in the near-wellbore zone and compatibility/injectivity has been verified—and when sequential or staged treatments are more appropriate, for example, when damage zones are spatially separated or component incompatibility cannot be avoided. Quantitative composition–performance relationships should be established, but reported synergy must be distinguished from proposed synergy. Most integrated three-function systems remain at the laboratory stage, and dynamic high-temperature/high-pressure core-flooding and field validation are required before practical benefits can be claimed.

(3) Construction of coupled static–dynamic evaluation systems

Future evaluation should integrate conventional static tests with dynamic core flooding, high-temperature/high-pressure displacement, microfluidic visualization, and wellbore simulation. Realistic brine, condensate oil, and porous media should be used to mimic deep reservoir conditions. Evaluation criteria should include not only anti-swelling rate, foam half-life, and contact angle but also dynamic adsorption retention, permeability recovery, liquid unloading efficiency, capillary number variation, and long-term stability. Such an evaluation framework will improve the reliability of agent screening and reduce the gap between laboratory results and field application.

(4) Green and low-cost synthesis processes

High-performance does not necessarily require high environmental cost. Future synthesis should focus on low-cost raw materials, simple reaction routes, scalable processes, and biodegradable products. Fluorine-free alternatives, bio-based surfactants, and degradable functionalized surfactants should be explored. Life-cycle assessment, toxicity testing, and biodegradability evaluation should be incorporated into material design. The goal is to balance application performance, environmental protection, and production cost, thereby enabling industrial promotion.

### 4.3. Concluding Remarks

The reviewed literature shows that deep gas reservoir damage can involve clay-related matrix damage, wellbore liquid loading, and pore-scale liquid trapping, which may occur independently or together. A universal condition-dependent sequence has not been established, which may spatially overlap and have been experimentally shown to be compatible, integrated or staged. Multi-function remediation is a promising research direction; it is not a universal requirement for agents for deep complex gas reservoirs. However, such integrated systems remain largely at the laboratory stage; their compatibility, injectivity, dynamic high-temperature/high-pressure performance, and field applicability require systematic validation before practical benefits can be claimed. Such agents may provide chemical technical support but remain largely at the laboratory stage. Such agents are required for efficient and stable deep natural gas production, to contribute to national energy security, and to promote the green and sustainable development of unconventional and deep gas resources.

## Figures and Tables

**Table 1:** Foaming performance of foam-drainage composite surfactant under different condensate oil concentrations (at 30 °C) [52].

Concentration/%	0 min/cm	5 min/cm	10 min/cm	15 min/cm	20 min/cm
5	21.3	21.7	21.7	21.3	21.0
10	21.5	21.6	21.5	21.5	21.1
20	21.6	21.9	21.5	19.4	16.7
30	21.9	19.8	16.3	12.7	8.1

**Table 2:** Summary of parameters and limitations of representative surfactant systems from the published literature [24,37,45,46].

Formulation	Name	Concentration/wt%	Temperature/°C	Press/MPa	Salinity/mg/L	Condensate Content	Test Method	Main Limitations
Clay stabilizers	Cationic hyperbranched polymer (HBP-HTC)	1	120	Atm	2 × 10^4^	0	Static anti-swelling test	Molecular chains prone to blocking low-permeability pores
Foam-drainage agents	XY-1 sulfonic-acid betaine	0.5	90	Atm	3 × 10^4^	15	Static foaming test	Insufficient salt and oil tolerance
	CAGB zwitterionic surfactant	0.6	85	Atm	2.5 × 10^4^	10	Static foaming test	Foam ruptures rapidly at high condensate content
Liquid-lock agents	Anionic alkylbenzene sulfonate	0.4	90	Atm	1.5 × 10^4^	N.R.	Interfacial tension/contact angle	Poor salt tolerance, prone to precipitation
	Long-chain alkyl oxamide	0.3	120	10	3 × 10^4^	N.R.	Core flooding/Contact angle	Performance drops significantly with condensate

## Data Availability

The original contributions presented in this study are included in the article. Further inquiries can be directed to the corresponding author.
